# Reducing deaths and injury from road traffic crashes through multisectoral collaboration

**DOI:** 10.11604/pamj.supp.2023.45.1.39638

**Published:** 2023-06-06

**Authors:** Mary Kessi, Deus Sokoni, Mary Kitambi, Daudi Katopola, Galbert Fedjo, Neema Kileo, Evelyn Murphy, Mapunda Maxmillan, Leticia Rweyemamu, Jones Jema John, Elibahati Akyoo, Zablon Yoti

**Affiliations:** 1 WHO Country Office, Dar es Salaam, Tanzania,; 2 Tanzania Police Force-Traffic Unit, Dar es Salaam, Tanzania,; 3 Ministry of Health, Dar es Salaam, Tanzania,; 4 National Institute of Transport, Dar es Salaam, Tanzania,; 5 Legal and Development Consultant, Dar es Salaam, Tanzania

**Keywords:** Road traffic injury, multisectoral collaboration, coalition

## Abstract

**Introduction:**

road traffic injuries are the eighth cause of mortality globally, killing about 1.35 million people and leaving more than 50 million others with permanent injuries and disabilities yearly. In Tanzania, the impact of road traffic crashes is still high despite a noticeable reduction in the number of associated injuries. This paper seeks to lay the foundation for promoting multisectoral actions and collaborations in dealing with public health concerns due to increased consequences caused by road traffic deaths and injuries

**Methods:**

in 2015, a multisectoral approach was adopted to implement a 5-year (2015-2020) road safety program that aimed to advocate for amendment of the Road Traffic Act of 1973, Chapter 168 Revised Edition 2002. A series of consultative sessions were held between government and non-state actors, including different committees formed to feed each other on the agenda. The program was implemented through the Ministry of Health and the Ministry of Home Affairs in collaboration with World Health Organisation and civil society organisations.

**Results:**

it has been noted that there is a direct relation with a set of combined policy-level interactions seeking to improve the legal environment for road safety. The program committee, civil society organisations, and parliamentarians’ forum were solicited as essential stakeholders in advancing policy reform. Together they conducted a series of consultative meetings, resulting in having a Bill tabled in the Parliament as a first draft. This informed policymakers and raised their attention to the magnitude of road traffic crashes and the country’s social and economic burden.

**Conclusion:**

efforts still need to be expanded to analyse the existing data to understand the extent to which risk factors contribute to road crashes, injuries, and deaths. There is a need to have a strong Government involvement in strengthening ownership and sustainability of any public health intervention, such as road safety.

## Introduction

Road traffic injuries are the eighth cause of mortality globally, killing about 1.35 million and leaving 50 million people seriously injured [1]. Pedestrians, cyclists, and motorcyclists make up 50% of all fatalities. Tanzania has the highest death rate of 32 per 100,000 compared to 26.6 per 100,000 of Africa and 18 per 100,000 globally [1,2]. According to statistics released by the police, the burden of road traffic death and injuries in Tanzania is still high [3]. In March 2010, the World Health Organisation (WHO) General Assembly passed Resolution 64/2551, proclaiming the Decade of Action for Road Safety 2011-2020, pledging to prevent millions of road traffic deaths and injuries. The Decade of Action recommended that countries build road safety management capacity, improve road infrastructure safety, develop vehicles’ safety, enhance road users’ behaviours, and improve the post-crash response [4]. In April 2012, the General Assembly approved resolution 260, encouraging Member States to adopt and implement national road safety legislation and regulations on five risk factors [5]. The risk factors are drinking and driving, speeding, seatbelt use, child restraints and use of helmets [6]. The resolution also encourages countries to improve enforcement mechanisms by having random breathalyser checks at checkpoints [6].

The second Decade of Action for Road Safety 2021-2030 was proclaimed by United Nations (UN) Resolution Number 74/299. The main goal of the second Decade of Action is to reduce road traffic deaths and injuries by at least 50% by 2030. It encourages Member States to ensure political commitment to improving road safety, developing, and implementing road safety strategies, plans, legislation, and regulations with the involvement of stakeholders from all sectors and levels of government [7]. The following shows the key milestones in an attempt to address road traffic deaths and injuries at the global level. 1) World report on road traffic injury prevention-2004; 2) global status report on road safety: time for action, 2009; 3) the first global ministerial conference on road safety was subsequently held in Moscow from 19-20 November 2009; 4) General Assembly resolution 64/2551 of March 2010 proclaimed 2011-2020 as the Decade of Action for road safety; 5) WHO global status report on road safety 2013: supporting a decade of action; 6) global status report on road safety, 2015; 7) launching of the Global Legislators forum, 2016; 8) the Global status report on road safety, 2018; 9) UN General Assembly in 2021 adopted resolution A/RES/74/299 “Improving global road safety”, proclaiming the Decade of Action for Road Safety 2021-2030.

The Government of the United Republic of Tanzania, through the Ministry of Health and the Ministry of Home Affairs, in collaboration with WHO and civil society organisations, implemented a 5-year road safety program using a multisectoral approach to advocate for amendment of the Road Traffic Act of 1973, Chapter 168 Revised Edition 2002. The program was funded by Bloomberg Philanthropies and ran from 2015 to 2020 aimed to control deaths and injuries by enacting effective road safety measures [8]. Tanzania implemented this program parallel to China, the Philippines, Thailand, and India. WHO and its partners provided technical assistance to analyse and revise existing laws. Also, it supported capacity building to a pool of lawyers to advocate for laws and regulations and capacitating journalists to write more in-depth stories about road safety dynamics and solutions [9]. A multisectoral approach was used to address policy reform on the five risk factors. This approach was adopted as a tested strategy and showed success in other countries’ attempts at mobilising efforts from stakeholders across sectors to collaborate to control non-communicable diseases [10]. Multisectoral action is vital if the country is to meet target 3.6 of the Sustainable Development Goals, aiming to reduce road traffic deaths and injuries by 50% by 2030 [11]. This paper seeks to lay the foundation for promoting multisectoral actions and collaborations in dealing with public health concerns due to increased consequences caused by road traffic deaths and injuries.

## Methods

**Setting:** the Road Safety Program was launched and implemented in Tanzania from May 2015 to December 2020. The program was implemented through multi-pronged approaches, working with stakeholders comprising government ministries, departments and agencies, civil society organisations, parliamentary committees, parliamentarians, media houses, and legal professional associations. The following approach was used:

Official launch of the program: following internal consultations between the WHO and the government over the process and timelines for project execution, the road safety initiative was launched in May 2015. The launch of the initiative for improving road safety took place in Dar es Salaam and included officials from both the government and non-profit groups. The program’s official inauguration generated momentum and made the intended audience, desired goals, and proposed techniques evident. The decision to establish a multisector project coordination committee, where the government and important stakeholders may meet and openly discuss the program, was made during the meeting. Using the program’s high visibility in the media, the program’s goal of reducing traffic fatalities through law change was well understood and expected.

**Creation of a National Program Coordination Committee:** WHO and the Ministry of Health facilitated the formation of a project coordination committee by the government to oversee the project implementation. Official communications were sent to relevant ministries and organisations to inform the management about the project and requested them to select one senior staff to be a member of the coordination committee. The committee incorporated 15 members from government institutions, civil society, academics, and WHO. Members of the committee were: the Ministry of Heath, Attorney Generals’ Chamber, Ministry of Transport, Ministry of Information Youth and Culture, Ministry of Justice and Constitutional Affairs, Ministry of Education and Vocation Training, National Road Safety Council, Ministry of Home Affairs, Surface and Marine Transport Regulatory Authority (SUMATRA) as it then was, Safe Speed Foundation, Helmet Vaccine Initiatives, Prime Minister’s Office, Ministry of Works, Ministry of Transport and WHO providing secretariat role. The terms of reference were developed to direct the coordination committee. Among the roles listed were preparing a work plan and guiding the legislative process. It also considered recruiting implementers for the program’s legal and media development, identifying key stakeholders, offering guidance on the mechanism for legal reform, and consulting with stakeholders. An in-depth stakeholder analysis was done to ensure that all interest groups were considered and consulted throughout drafting of the road traffic amendment. The committee scheduled monthly meetings during the first year to discuss project developments and approve plans for the upcoming month. Later, the group decided to hold quarterly meetings because most project operations had already started and went smoothly.

**Media and legal assessments:** media and legal assessments were conducted to identify strengths, gaps, and areas that need improvement. The assessments were intended to show the situation at the beginning of the program interventions to establish the benchmarks for success, especially regarding the identified key risk factors. Concerning the legal assessment, Tanzania’s road safety legal and policy framework was found to contain gaps in drinking and driving provisions, speed restrictions, and use and non-use of seatbelts or child restraints for all passengers in motor vehicles. Also, gaps related to the use and non-use of helmets for cyclists and riders of motorised two-wheelers and setting blood alcohol limits for drivers. The following gaps were identified: seat belt: Section 39 [11] of the Road Traffic Act Cap 168 Revised Edition (RE) 2002 requires only the driver of motor vehicles and front seat passengers to wear a seat belt [12]. Helmet: Section 39 [12] of the Road Traffic Act requires only a person who drives a two or three-wheeled motorcycle to wear a helmet. Changes have allowed motorcycles to be used for commercial purposes, but the provisions on helmet use do not cover the use of helmets for passengers. The helmet standard is not addressed in the law [13]. Drinking and driving - blood alcohol concentration limit under the Tanzania Road Traffic Act, the drinking and driving limit is set at 0.08g/dl, which is higher than the recommended international level of 0.05g/dl. Speeding: the speed limit in the built-up area is set at 50 kilometres per hour, and the speed of a vehicle outside a built-up area is regulated according to traffic signs and road markings. Similarly, the legislation does not address the issue of child restraint [13].

The main challenges are multiple laws and regulations providing for the same matter. For example, the Roads Act, 2007 establishes the Tanzania National Roads Agency (TANROADS); and empowers the Minister responsible for road construction and transportation to regulate road safety generally and matters relating to speed specifically with the view of protecting road quality and safety. Under the general classification of the road network in Tanzania, all national roads and trunk roads fall under the mandate of TANROADS. Since establishing the Tanzania Rural and Urban Roads Authority (TARURA), which came into force in 2017, the regional and district roads, the class of roads that formerly had been under the Local Government Authorities (LGAs), have been taken over by TARURA. TARURA is now responsible for constructing and maintaining roads, so it has the mandate to manage speeds at the local level. All these mean that the concept of road safety and statutory power to dictate the standards is shared across different government agencies. For example, the Road Traffic Act, Cap 168, RE 2002, the Roads Act, 2007, and the Transport Licencing Act, Cap 37, RE 2002 have respectively mandated the Ministry of Home Affairs, the Ministry of Works and Transport through TANROADS and TARURA; and Land Transport Regulatory Authority (LATRA) with the responsibility of regulating land transport matters including safety. Relating to media assessment, it was revealed that the Tanzanian media focuses primarily and exclusively on traffic-related incidents as events crash reporting rather than road safety reporting, emphasising the carnage on the country’s roads. Coverage of road safety showed an absence of understanding that traffic crashes occur because of a complex set of social, cultural, economic, and regulatory determinants, which indicates a significant knowledge gap in the media. Further, the media was not focused on educating the public and were prone to using scare tactics to highlight the horrific numbers associated with deadly crashes, which often included gruesome images [14]. Road safety as a theme was only ever reported on during the annual national road safety campaigns. It was recommended that there is a critical need to shift attitudes and behaviour among Tanzanians, given the overriding prevalence of human errors leading to fatalities and injuries on the country’s roads. It was agreed that the media has a role in helping the audience understand that road safety is more than just a police matter. Likewise, news managers, editors, and media owners are pivotal to the success of media programs [15]. It was also revealed that everyone should view road safety as a public health concern rather than falling squarely into the public realm.

**Formation of Civil Society Coalition for Road Safety:** knowing the importance of advocacy by civil society in any legal reform, the WHO supported the formation of the Civil Society Coalition for Road Safety. The coalition for road safety amendment was formed together with a technical steering committee coordinated by Tanzania Women’s Lawyers Association (TAWLA). The coalition has a total of 16 Civil Society Organisations. The following are the coalition members: Tanzania Women Lawyers Association, Tanganyika Law Society, Road Safety Ambassadors, Mwanza Youth and Children Network, Tanzania Women in Media, and Tanzania Child Rights Forum. Other members are the Safe Speed Foundation, Women in Law and Development, Tanzania Media Foundation, Media Space, Tanzania Federation of Disabled People’s Organisations, Sikika, and Tanzania Bus Owners Association. The National Institute of Transport also joined the coalition. The coalition developed a joint position paper on Road Traffic Act Amendments. It carried out advocacy activities targeting policy makers and behaviour change activities targeting road users to contribute to the coalition strategy. The coalition received funding for organising activities and prepared a coalition position paper. The position paper identified all the legal gaps and issued recommendations on the laws’ provisions that needed to be changed. Several meetings and training were conducted for coalition members, road safety technical staff from relevant ministries, journalists, lawyers, and members of Parliament.

**Establish and implement a Journalist Fellowship Program:** the Road Safety Media Fellowship was implemented first through the Tanzania Media Foundation (TMF) and later by the program coordination committee. The program aimed to address legal gaps in the Tanzania Road Safety laws and create awareness of road safety risk factors by engaging media houses and individual journalists in reporting comprehensive and quality road safety stories. The objective of the Media Fellowship was to increase the number and improve the quality of news reporting and create impactful stories on road safety to increase public awareness and agitation for the legal reform that would lead to adopting evidence-based laws and regulations in the country. The program involved active participation and practical application, emphasising WHO actions and the plateau in global road safety. Additionally, the program emphasises domestic policy, legal strengths and weaknesses, practical news pitches for road safety, investigative journalism, ethical journalism, and the persuasive force of media. By 2020, the program trained 100 journalists in reporting stories that would bring impact. Each fellow produced a minimum of six well-investigated stories and two blog stories. Fellows were actively involved in stakeholders’ consultations and advocacy with civil society organisations such as Tanzania Media Women Association (TAMWA) and Tanzania Women Lawyers Association (TAWLA).

Implementation of a Legal Development Program (LDP): formation and implementation of a Legal Development Program (LDP) through which 35 government, university, and civil society lawyers participated. The objective of the LDP was for its members to advocate for and contribute to the drafting and adoption of evidence-based laws and regulations in the country. The LDP implementation of capacity-building activities aimed at improving the understating of road safety (including risks and interventions to minimise those risks), road safety laws, and international best practices for lawyers, civil society members, and journalists. As part of the LDP in 2017, 10 lawyers from academia, civil society, lawyers’ professional bodies, and government ministries and departments were engaged in discussions at national and international levels on road safety legislation. The lawyers developed proposals for a legal framework for road safety in Tanzania through national consultations on road safety. They also worked closely with media, civil society organisations, coalitions, and other partners to elaborate on the position of the law, the risks, and ongoing interventions to improve the legal framework. The LDP aimed to facilitate the development of evidence-based advocacy on laws and regulations on road safety as well as building and strengthening a professional network in the field of road safety.

**Engaging parliamentarians in Road Safety for political leadership:** the Program Committee and civil society also mobilised parliamentarians as essential stakeholders in advancing policy reform. They can assist in formulating effective national laws and regulations for road safety. They can also contribute to making a case for increased budget allocation for road safety sector financing [16]. Similarly, parliament members can provide injury prevention oversight to public and governmental accountability authorities on their pledges to promote road safety. Following the establishment of the Global Network for Road Safety Legislators in 2016, it was deemed essential to emulate the same effort to cascade the global initiative to the individual countries’ level. This would create a team of dedicated Members of Parliament who were adept in road safety and serve as an extended arm of the campaign within Parliament [16]. Thus, the Members of Parliament Coalition was established in 2017 to spearhead the call for an amendment to the Road Traffic Act and advocate road safety awareness campaign within Tanzania Parliament.

In collaboration with the Civil Society Coalition for road safety legal reform, WHO organised roundtable discussions with the members of Parliament. Hon. Adadi Rajab (MP), who was nominated to attend the launching of the Global Legislatures Forum, presented feedback from the meeting. The main objective was to make Members of Parliament sign up for the Global Forum for Road Safety Legislators Westminster Declaration in December 2016. As a result, 110 members of Parliament signed a declaration to support road safety reform and the formation of a network of legislators. A committee was established to prepare for the legislator’s association on road safety registration to speed up the process. WHO, along with the National Coordination Committee, continued engagement with the Parliamentarians by holding briefing sessions in Dodoma on why the Road Traffic Act amendment was needed, the content of the amendment, and additional improvements. It was proposed that the briefing be held during the Parliamentary Committee’s sessions to reduce the cost of inviting them from their constituencies which would have been more expensive. The establishment of the Members of Parliament Coalition in 2017 spearheaded the call for an amendment to the Road Traffic Act. It advocated a road safety awareness campaign in the Tanzania Parliament.

**Drafting of the bill for amendment:** a series of consultative sessions were held among government and non-state actors’ stakeholders, including the different committees formed to feed each other on the agenda. This also provided a form of backstopping support, tapping on each other relative strengths when meeting the statutory committees such as the official parliamentary committees and the higher rungs of the government policy-making structures. Eventually, the Ministry of Home Affairs, in collaboration with the Office of the Attorney General and stakeholders, developed a draft bill of amendment for road safety to address the risk factors. The drafted bill of amendment of the Road Traffic Act was tabled in the Parliament as a first reading, which awakened the attention of policymakers to the magnitude of the road traffic injury to social and economic burden.

**Legal consultative sessions:** through the Legal Development Fellowship Project, consultative sessions were held that focused more pointedly on matters of legal intricacies, bringing together lawyers, police officers, magistrates, prosecutors, and advocates to build a shared understanding of the status of road safety and laws in Tanzania. During these consultations, lawyers in different portfolios and backgrounds shared best practices. They provided input on the nature and form of the changes to be effected to the Road Traffic Act and other associated legislation. The consultations helped bring up the often-ignored expert experiential knowledge based on the practitioners’ perspectives. Such consultations developed and sharpened the depth and breadth of the reform agenda in a meaningful and informed way.

**Data intervention:** the exercise for data collection was conceived as a parallel output area for countries implementing the road project globally. This had been designed to improve data collection mechanisms at the country level, especially building from the gaps identified during the biennial Global Report on Road Safety preparations. This is a flagship report by the WHO, a UN agency mandated by various United Nations General Assemblies Resolutions to generate such information and data; and lead the global efforts in articulating research and reforms relating to road safety globally. As part of implementing the national program, the Ministry of Home Affairs, mandated by the Road Traffic Act, Cap 168 RE 2002, had to be supported to design a strategy to improve the road traffic’s data collection. The main objective was to improve the quality and integrity of collected road traffic crash data while augmenting future global road safety reports. Over-dependency on police data had created a set of problems of its own and therefore been brought into sharp disrepute regarding their accuracy, promptness, and overall reliability. It had been noted in earlier assessments that data from police were grossly inadequate at best. The police have proved to have had an overwhelming focus on collecting the frequency of the crash incidents’ data that shows the where, when, what, and who. Additionally, they were inadequately analytical, which limited the ability to generate good analyses to inform the global report.

In an ideal situation, the National Road Traffic Commander and the National Road Safety Council are essential sources of road safety data in Tanzania. For example, while the Traffic Commander is vested with the duty to produce the national statistics and disseminate the same to the public, the National Road Safety Council’s role is to conceive of the research in road safety practices and disseminate the generated knowledge. Against this backdrop, the project acknowledged the existing gaps in the coordination of data and accepted that it could at least try and work within the premises of the current constraints. Thus, the National Coordination Committee agreed that the Traffic Police unit is assisted in collecting better versions of the datasets assisted technically by the Ministry of Health, the Ministry of Works and Transport, and other national-level statistics agencies recognised as such. This is in keeping with the impetus provided by the UN Decade of Action for Road Safety 2011-2020. The Decade of Action encourages countries to enact road safety laws to conform to international standards to achieve specific regulatory and institutional outcomes. Also, achieving those outcomes could improve coordination by designating the lead agency. They should also have basic, freely available, independently verifiable data emerging from sufficiently credible sources and analysed with such rigour to provide evidence for the policy reform agenda and overall improvement of road safety in the country.

**Types and sources of data used:** with the authorisation and collaboration with the Traffic Police unit, Data in MS Excel format were collected and analysed by the Tanzania Police Force (Traffic Unit). Project reports from WHO and from civil society were also reviewed, and the data analysed from the police were from 2012-2021. The main objective was to assess the trends of road traffic deaths and injuries in the country and to see whether the country is making progress in reducing the number of people injured or killed in road traffic crashes. We obtained an in-depth analysis of why road users’ behaviours remain to be seen and may be subject to independent assessment. The nature and type of data are recorded immediately after reporting the occurrence of a crash and captured in the Police Incident Report Book. The Primary Source of verification, therefore, is solely the Police Incident Report Book. Data still broadly captures the who, where, when, and what. Connecting to this, however, is the ongoing implementation of the Road Accident Information System (RAIS) currently held by the Ministry of Works and Transport.

## Results

The tabling of the Road Traffic Act Amendment Bill for the first reading on June 30, 2021, resulted from joint efforts from the government and non-state actors. The government agreed to amend the Road Traffic Act and submitted to the Parliament a bill of the amendment. The proposed legislative changes address four of the five risk factors advocated by the government and civil society. For example, the amendment required all vehicle occupants to wear seatbelts. Further, Section 39 D [3] calls for mandatory child restraints, and section 49 reduces the blood alcohol limit from 0.08 g/dl to 0.05 g/dl [17].

**Trends and patterns by project objective**

**Figure 1:**
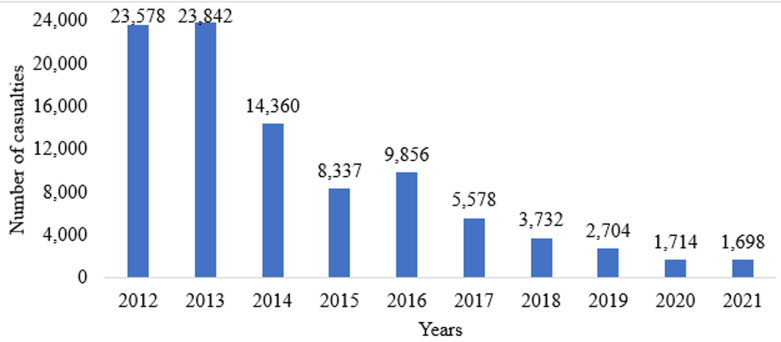
trend of road crashes in Tanzania from 2012 to 2021

Figure 1 indicates the progressive trend towards reducing road traffic casualties resulting from road crashes. Since 2012, the number of causalities has been decreasing in Tanzania. In 2021, there has been a decrease of about 92% in casualties compared to the 2012 level in the same category.

There is a significant reduction in injuries compared to those recorded in 2012 by 90%. Table 1 shows that Tanzania has recorded a slight decline in road traffic deaths and injuries from 3,966 deaths and 20,533 injuries in 2013 to 1,240 deaths and 2,019 injuries in 2021, according to data collected by the police force. Likewise, Table 2 depicts a steady decrease in death from road crashes compared to 2012 by 61%.

**Table 1:** deaths and injuries for different road users in Tanzania from 2012-2021

Categories of road users	2012		2013		2014		2015		2016	
	Death	Injuries	Death	Injuries	Death	Injuries	Death	Injuries	Death	Injuries
Drivers	293	1364	297	1528	280	1000	236	629	266	643
Passengers	1185	8477	1105	8770	1222	6790	1117	4621	988	4593
Motorcyclist	808	4939	870	5237	795	3359	828	2106	739	1866
Cyclists	405	1240	447	1162	343	579	281	319	261	242
Pedestrians	1212	3909	1247	3836	1103	2739	978	1681	975	1598
**Total**	3903	19929	3966	20533	3743	14467	3440	9356	3229	8942
**Categories of road users**	**2017**		**2018**		**2019**		**2020**		**2021**		
	**Death**	**Injuries**	**Death**	**Injuries**	**Death**	**Injuries**	**Death**	**Injuries**	**Death**	**Injuries**
Drivers	202	411	178	352	153	280	151	183	168	175
Passengers	790	3157	707	2035	469	1608	502	1355	455	1436
Motorcyclist	623	963	313	648	309	437	236	288	254	204
Cyclists	204	124	114	88	111	55	65	30	59	22
Pedestrians	751	820	467	593	381	450	311	265	304	182
**Total**	2570	5475	1779	3716	1423	2830	1265	2121	1240	2019

**Table 2:** deaths rate of various road users from 2012-2021 in Tanzania

Row Labels	2012 death rate	2013 death rate	2014 death rate	2015 death rate	2016 death rate	2017 death rate	2018 death rate	2019 death rate	2020 death rate	2021 death rate
Drivers	21%	19%	28%	38%	41%	49%	51%	55%	83%	96%
Passengers	14%	13%	18%	24%	22%	25%	35%	29%	37%	32%
Motorcyclist	16%	17%	24%	39%	40%	65%	48%	71%	82%	125%
Cyclists	33%	38%	59%	88%	108%	165%	130%	202%	217%	268%
Pedestrians	31%	33%	40%	58%	61%	92%	79%	85%	117%	167%
Total	20%	19%	26%	37%	36%	47%	48%	50%	60%	61%

Table 3 shows number decrease and increase of death while Table 4 shows number decrease and increase of injuries among drivers, passengers, motorcyclists, cyclists, and pedestrians from 2013 -2021 in Tanzania mainland. The most affected group is the passengers, followed by motorcyclists and pedestrians. Passengers still constitute the largest group of affected road users compared to the rest of the categories of road users, such as drivers, followed by motorcyclists, cyclists, and pedestrians throughout the years under review. It can also be observed from the tables that motorcyclists are faring a little better than pedestrians in 2020 and 2021.

**Table 3:** changes in the number of deaths amongst road users in Tanzania from 2012-2021

Road Users	2012/2013	2013/2014	2014/2015	2015/2016	2016/2017	2017/2018	2018/2019	2019/2020	2020/2021
Drivers	4	-17	-44	30	-64	-24	-25	-2	17
Passenger	-80	117	-105	-129	-198	-83	-238	33	-47
Motorcyclist	62	-75	33	-89	-116	-310	-4	-73	18
Cyclists	42	-104	-62	-20	-57	-90	-3	-46	-6
Pedestrians	35	-144	-125	-3	-224	-284	-86	-70	-7
**Total**	143	-340	-198	-82	-461	-708	-118	-191	22

**Table 4:** changes in the number of injuries amongst road users in Tanzania from 2012-2021

Road Users	2012/2013	2013/2014	2014/2015	2015/2016	2016/2017	2017/2018	2018/2019	2019/2020	2020/2021
Drivers	164	-528	-371	14	-232	-59	-72	-97	-8
Passengers	293	-1980	-2169	-28	-1436	-1122	-427	-253	81
Motorcyclist	298	-1878	-1253	-240	-903	-315	-211	-149	-84
Cyclists	-78	-583	-260	-77	-118	-36	-33	-25	-8
Pedestrians	-73	-1097	-1058	-83	-778	-227	-143	-185	-83
**Total**	604	-6066	-5111	-414	-3467	-1759	-886	-709	-102

## Discussion

This paper extends the understanding that road safety management programs must be inclusive and adopt a multisectoral approach during development and implementation. It provides a broad discussion on the role of WHO and other stakeholders in implementing the program and influences the review of the Road Traffic Act, 1973. Throughout the paper, the enforcement component has been insisted on, and efforts to strengthen the National Road Safety Council have been pointed out. Also, the paper explored key milestones completed from stakeholders´ engagement, review sessions and reading of the bill for the first time in the Parliament. It is essential to keep the program’s objectives as one that seeks to implement the aims of the UN Decade of Action (UNDOA) 2011-2020. The Decade of Action had one of its targets to reduce road traffic deaths and injuries by at least 50% by 2030. If confirmed for its credibility, the emerging data at Tanzania’s country-level aligns with the global aspiration, which is a positive step. These results relate to a set of combined policy-level interactions seeking to improve the legal environment for road safety. Moreover, different program interventions implemented from 2015 to 2020 enabled the engagement of country-level stakeholders who conduct campaigns to raise road safety awareness. Similarly, the Annual National Road Safety Week has provided a platform for maintaining political momentum. Working with law enforcement in many aspects of strengthening the enforcement mechanism was one of the pillars of the UNDOA. Thus, the identified gaps in the enforcement mechanisms have been brought to focus and addressed. Some are still pending the passing of the laws, but there is already momentum building to change practices even where such regulations are still absent. One such area is the insistence by the police to encourage road users travelling as passengers on motorcycles to wear helmets and all passengers in the public service vehicles plying intercity to wear safety seatbelts. Awareness is also rising about the use of child restraints. Another area is increased attention to enforcing the drink and drive provisions of the Road Traffic Act.

In addition to observing the positive side, there is renewed energy toward strengthening the coordination mechanism by building the capacity of the National Road Safety Council established under the Road Traffic Act, Cap 168, RE 2002 [12]. Further work on this will be addressed by the pending amendment to RTA given the strong recommendations submitted. Civil society coalition for road safety and parliamentarians’ road safety network became an important forum for engagement to discuss the need for legal reform. This is because when the civil society coalition conducted training and legal awareness session, parliamentarians were tasking the Government by asking road traffic crash questions during the Parliament’s sessions. This has created a push factor for the police enforcers, knowing that there is big brother watching whatever they do. Journalists’ and lawyers’ engagement has supported the increased public awareness of the magnitude of road traffic crashes in the country and their impact. On the other hand, the study relied on data from the Traffic Police database, the country’s only reliable source of traffic data. The database could be incomplete due to the possibility of misreporting or underreporting crash information. However, it is the only reliable source available in the country, though it could have been enriched by using hospital data. Also, the program could have been affected by the multitude of stakeholders influencing the functioning of road safety initiatives in Tanzania. For example, editors and media owners were involved into the program to ensure sustained media coverage of road safety from a public health perspective. Although they are not directly writing articles, their ownership of the program was vital. So, the identification of leading stakeholders among coalition members and regular communications enabled the effective implementation of the program.

## Conclusion

Although the number of deaths is being reduced with the reduction of causalities, there is still a need to see the link between ongoing interventions and improved post-crash care results. Efforts still need to be expanded to analyse the existing data to understand how drunk driving contributes to road crashes, injuries, and deaths. More significantly, more efforts are needed to save more lives by improving access to emergency services and managing cases. For effective collaboration coordination, clear target, agenda-setting, and communication with all stakeholders (i.e., government, law enforcers, Parliament, and non-state actors) are essential to address any public health concern. Joint consultation to identify gaps and agree on action to address them clarified the agenda to stakeholders. Also, strong Government involvement is needed to have ownership and sustainability of any public health intervention.

### What this study adds

- Road safety interventions are complex and involve multiple stakeholders;
- Government involvement is key in strengthening ownership and sustainability of any public health intervention, including road safety.

### What is known about this topic

- A set of combined policy-level interactions seeking to improve the legal environment for road safety are linked together;
- Essential stakeholders such as program committee, civil society organizations and parliamentarians were solicited in advancing policy reform.
